# Local thrombolytic therapy in acute mesenteric ischemia

**DOI:** 10.1186/1749-7922-8-8

**Published:** 2013-02-09

**Authors:** Fatih Yanar, Orhan Agcaoglu, Inanc Samil Sarici, Emre Sivrikoz, Adem Ucar, Hakan Yanar, Murat Aksoy, Mehmet Kurtoglu

**Affiliations:** 1 Istanbul Medical Faculty, Department of General Surgery, Istanbul University, Istanbul, Turkey; 2 Istanbul Medical Faculty, Department of Radiology, Istanbul University, Istanbul, Turkey; 3 Department of General Surgery, Bahcesehir University, Istanbul, Turkey

**Keywords:** Acute mesenteric ischemia, Thrombolysis, Laparoscopy, Second-look, CT-Angiography

## Abstract

**Background:**

The aim of the study was to evaluate the local thrombolytic therapy (LTT) in combination with laparoscopy, in management of acute mesenteric ischemia (AMI).

**Methods:**

From January 2000 to January 2010, patients who were admitted to the hospital with AMI due to acute arterial occlusion were analysed retrospectively. Patients presenting with acute abdomen with a suspicion of AMI were evaluated with computerized tomography angiography (CTA). Patients who had findigs of AMI on CTA, were underwent selective mesenteric angiography and LTT eventhough without peritoneal signs. LTT was carried out before or after laparoscopy or laparotomy, and initiated with recombinant plasminogen activator.

**Results:**

LTT was performed in 13 (17.1%), out of 76 patients. From the remaining patients, 56 underwent necrotic bowel resection and 7 underwent tromboembolectomy. The median age was 62 years (45–87). The median duration of symptoms was 24 h. Four (30.7%) patients presented within 24 h onset of symptoms, whilst 9 (69.3%) patients presented after 24 h onset of symptoms. There were 5 (39.5%) patients, who presented with abdominal pain without peritoneal signs on physical examination and 8 (61.5%) patients, who had peritoneal signs. The mortality rate was 20% (1/5) in the first group who presented without peritoneal signs, whilst it was 62.5% (5/8) in the remaining.

**Conclusion:**

Early intervention in AMI is the key to better results. CTA combined with early laparoscopy and LTT may have beneficial effects at this setting.

## Introduction

Acute mesenteric ischemia (AMI) is a lethal disease with high mortality rates ranging from 24 to 94%. This is attributed to delayed diagnosis, ineffective treatment regimens and moribund patients [1-3]. Recent advances include use of computerized tomography angiography (CTA) for prompt diagnosis, sophisticated methods such as local thrombolytic therapy (LTT) for treatment and laparoscopy for both. Multidetector CTA is a fast and accurate method with a sensitivity and specificity of 94 and 96%, respectively [4,5]. This diagnostic accuracy has been combined with promising treatment alternatives, mainly LTT, and better prognosis has been achieved [6,7]. Recently, laparoscopy has proved itself as an evaluation method of acute abdomen. Thus, laparoscopic exploration became available for diagnosis of necrotic bowel segments, and treatment strategies are tailored thereafter [8]. Second look laparoscopy in order to assess bowel viability after bowel resection or thrombolysis has been employed frequently, which further improves outcomes in acute mesenteric ischemia [9].

This paper aims to evaluate the experience of a referral center in acute mesenteric ischemia and results of the algorithm applied.

## Materials and methods

From January 2000 to January 2010, patients who were admitted to the hospital with AMI due to acute arterial occlusion were analysed and records and data charts of all these patients were evaluated retrospectively.

**Figure 1:**
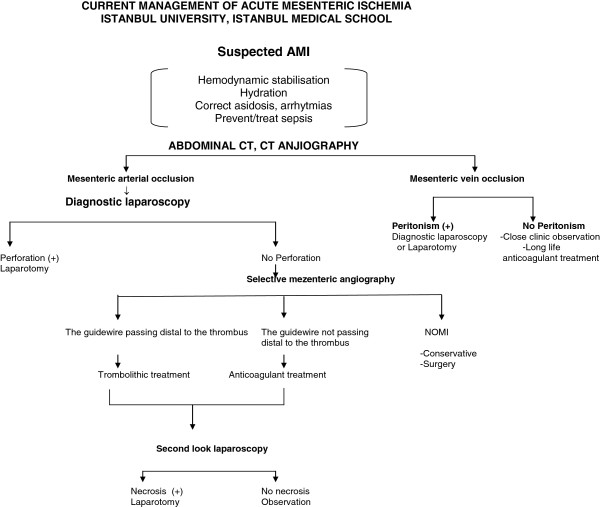
The algorithm applied during the study period covered diagnosis and treatment of AMI.

The algorithm applied during the study period covered diagnosis and treatment of AMI (Figure 1). Patients presenting with acute abdomen with a suspicion of AMI were evaluated with CTA. Patients, who had findings of AMI on CTA, without peritoneal signs selective mesenteric angiography and LTT were commenced. Should these patients develop peritoneal signs during treatment, surgical exploration (preferably laparoscopy) was undertaken. If peritoneal signs were positive during admission, laparoscopy was performed to assess bowel viability. If necrotic bowel segments were found, intestinal resection with anastomosis or enterostomies was performed and a second look procedure was planned after 24 h. In patients with critical bowel ischemia or partial salvageable bowel segment, either surgical or endovascular revascularization, namely LTT was carried out. The port positioned for laparoscopy post laparotomy to right lower quadrant and due to the timing of second look procedure, which was between 48 to 72 h, the previous skin incision had already totally sealed airtight on its own.

The method of mesenteric angiography included lateral aortography and catheterization of SMA. The guidewire was threaded into the orifice of the artery. If the SMA could be catheterized, LTT was initiated with recombinant plasminogen activator (rt-PA, Actilyse®, Boehringer Ingelheim GmbH) of 5 mg bolus, followed by 1 mg/h maintenance. After 24 h of treatment another angiography was performed and the catheter was withdrawn.

Patients were discharged with anticoagulating therapy in cases of AMI due to emboli and antiplatelet therapy and statins in cases of atherosclerosis and at follow-up they were evaluated with CTA at 3^rd^, 6^th^ and 1 year.

## Results

LTT was performed in 13 (17.1%) patients. From the remaining patients, 56 underwent necrotic bowel resection and 7 underwent tromboembolectomy. The median age was 62 years (45–87). There were 11 (84.6%) males and 2 (15.4%) females. All patients presented with acute abdominal pain. There were no patients with a known diagnosis of chronic mesenteric ischemia (CMI). However, history revealed post-prandial pain suggestive of CMI in 3 patients (23%). The median duration of symptoms was 24 h. Four (30.7%) patients presented within 24 h of onset of symptoms, whilst 9 (69.3%) patients presented after 24 h of the onset of symptoms. Diabetes mellitus was present in 8 (61.5%), hypertension in 6 (46.1%), hyperlipidemia in 2 (15.3%) patients, ischemic heart disease in 7 (53.8%), smoking in 7 (53.8%), and arythmia in 6 (46.1%) patients. Physical examination revealed positive peritoneal signs in 8 (61.5%) patients, while there were not any physical findings in 5 (39.5%) patients.

Patients without peritoneal signs on physical examination and with AMI findings on CTA underwent percutaneous SMA catheterization and LTT. One patient had multiorgan failure during the treatment and died. There were not any signs of intracranial or internal bleeding during the hospitalization of the patient. All other four patients improved and discharged without any further intervention and followed-up by CT- angiography on 3^rd^, 6^th^ and 1 year follow-up. The admission time was less than 24 h in four of these patients.

**Figure 2:**
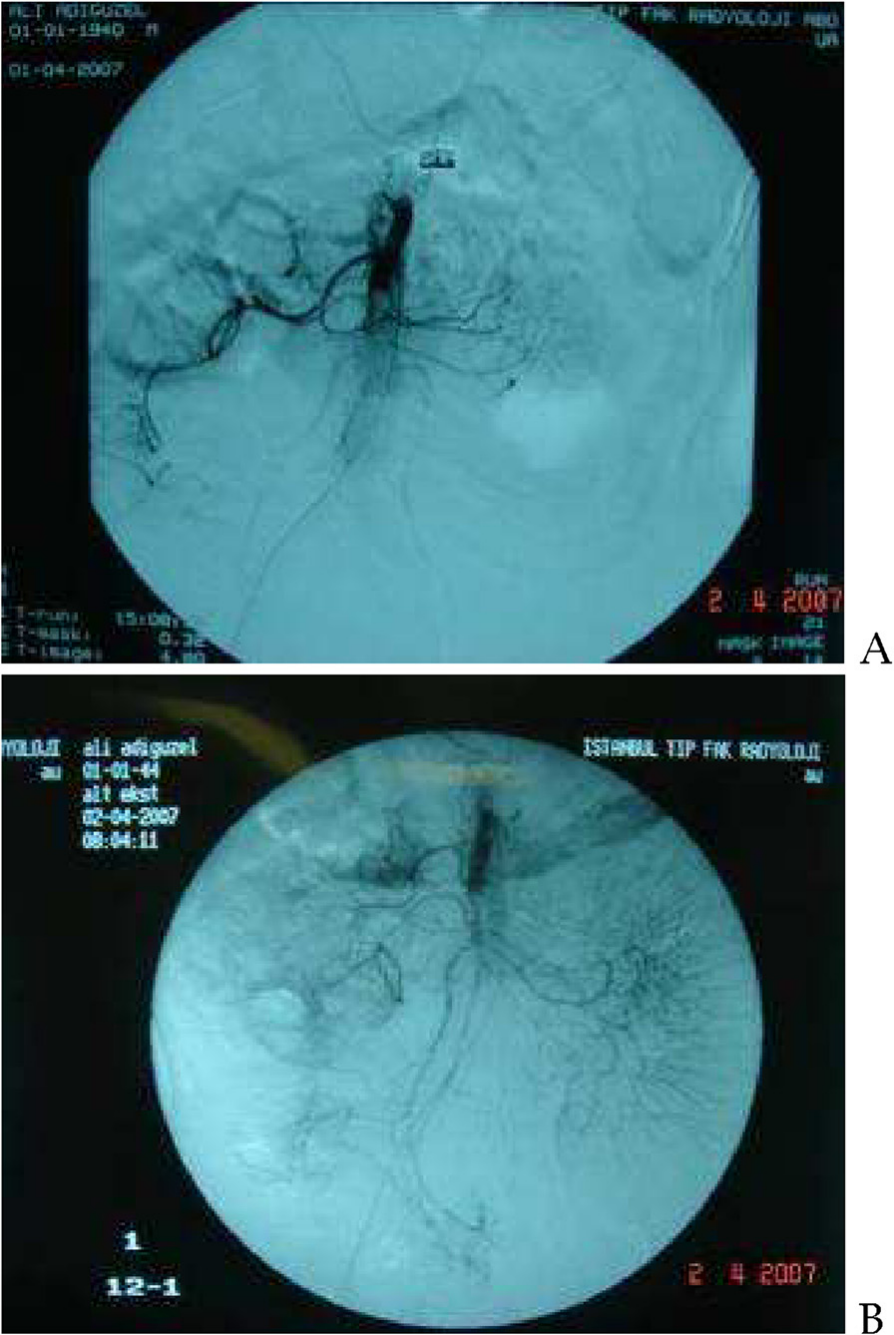
24-h digital subtraction angiography control reveals an improved mesenteric circulation (A) when compared to images obtained before local thrombolytic therapy (B).

There were 2 (15.3%) patients, who presented with peritoneal signs. One of the patients had findings of AMI on CTA. Both patients underwent laparoscopy. Low-flow state without bowel necrosis was positive during the evaluation. Percutaneous access to SMA was achieved and LTT was commenced. After 24 h, a control digital subtraction angiography was performed and revealed recanalization of SMA (Figure 2). There were no signs of peritoneal irritation in these patients; therefore second-look laparoscopy was not planned.

**Figure 3:**
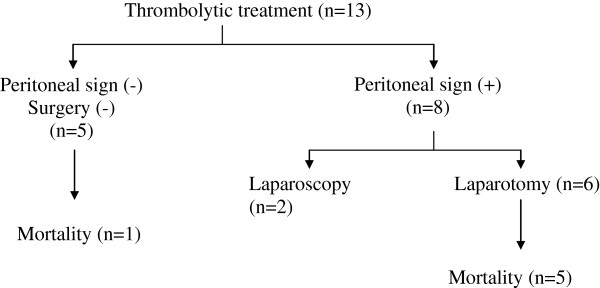
The mortality rates according to algorithm are shown.

There were 6 (46.1%) patients, who presented with peritoneal signs. One of the patients had findings of AMI on CTA. He underwent laparoscopy and subsequently laparotomy when positive findings for possible bowel necrosis were revealed during laparoscopy. However, there was not any bowel necrosis and the patient did not undergo bowel resection. He was then referred to LTT. A second-look laparoscopy was performed and there was not any further intervention. The patient died on day 5 of his hospitalization due to myocardial infarction. Three of these patients underwent laparotomy for acute abdomen and AMI was diagnosed during the exploration. Partial bowel resection without extensive dissection was carried out and the patients were then referred to LTT. Second-look laparotomy was associated with partial bowel resection. One of these patients had to undergo a third intervention for anastomosis leakage and enterostomy was carried out. The patient died of sepsis at the intensive care unit. The other two patients died with septic shock on day 10 and 12 after the first intervention. There were two other patients, who underwent laparotomy and AMI was diagnosed during exploration. Partial bowel resection was carried out in these patients and a laparoscopic port was placed for subsequent second-look. The patients received local thrombolytic therapy. In one of them, second-look laparoscopy revealed partial bowel necrosis and required partial bowel resection. The patient did not require any further intervention. The second-look laparoscopy for the last patient revealed normal findings, and he did not require any further intervention. He died with myocardial infarction on day 7. The mortality rates according to algorithm are shown in Figure 3. There were no bleeding complications with these 6 patients, who underwent a surgical intervention and LTT.

## Discussion

Acute mesenteric ischemia is a potentially lethal disease. Early recognition and accurate intervention remains the cornerstone of treatment. Patients may present with severe abdominal pain despite mild physical signs. Therefore, clinical suspicion is mandatory for the diagnosis, though these findings may be absent in 25% of cases [10]. In this series, all patients presented with abdominal pain. However, symptoms ranged from mild to severe such as acute abdomen.

Duplex ultrasonography accurately identifies high-grade stenoses of the celiac artery and superior mesenteric artery (SMA), and is the diagnostic modality of choice for chronic mesenteric ischemia. However, it is not suitable for diagnosing acute arterial mesenteric ischemia. It is operator-dependent and overall diagnostic accuracy may change, especially at off-hours. Moreover, solely the proximal segment of SMA can be evaluated by duplex because SMA emboli tend to lodge more distally. This creates the potential for a false-negative result [11]. Furthermore, although there are case reports concerning contrast-enhanced ultrasonography in AMI, acute cases usually present with overt abdominal gas and inflammatory changes, which may intervene with imaging by duplex [12]. Therefore, recent advances in optimizing CTA had promising results in diagnosing AMI. Helical, multidetector and multislice CTA is a fast and accurate investigation for the diagnosis of acute mesenteric ischemia [13]. It delineates vascular anatomy, evaluates bowel necrosis and allows early diagnosis. In most cases CTA can be used as a sole diagnostic procedure with 96% sensitivity and 94% specificity [4,5]. In our patients, we preferred to use CT as a first diagnostic step.

Laparoscopic surgery, with high diagnostic accuracy can be safely and effectively applied to the patients with acute abdominal emergencies. It is a valuable tool to prevent unnecessary laparotomies when routine investigations fail to identify the cause. It provides a highly important advantage for detecting the degree of bowel ischemia in AMI following diagnosis with CTA [8]. Although its use in AMI is questioned in a recent review, our experience proved otherwise [14]. After laparoscopy has been successfully introduced and adapted for daily use over the years, its accuracy has been better by improving through technology [9]. Therefore, we utilize laparoscopic exploration in a routine basis in recent years and have shifted our treatment algorithm for AMI in favor of initial laparoscopic exploration. However, if the exploration can not provide enough information regarding the viability of the entire bowel, laparotomy is indicated.

Thrombolytic therapy is an effective and quick treatment modality for AMI and may obviate surgery and has the potential to resolve the clot completely [15,16]. If resolution occurs partially, it already serves as an adjunctive to surgery by sparing an amount of near-ischemic bowel segments [6,7].

**Figure 4:**
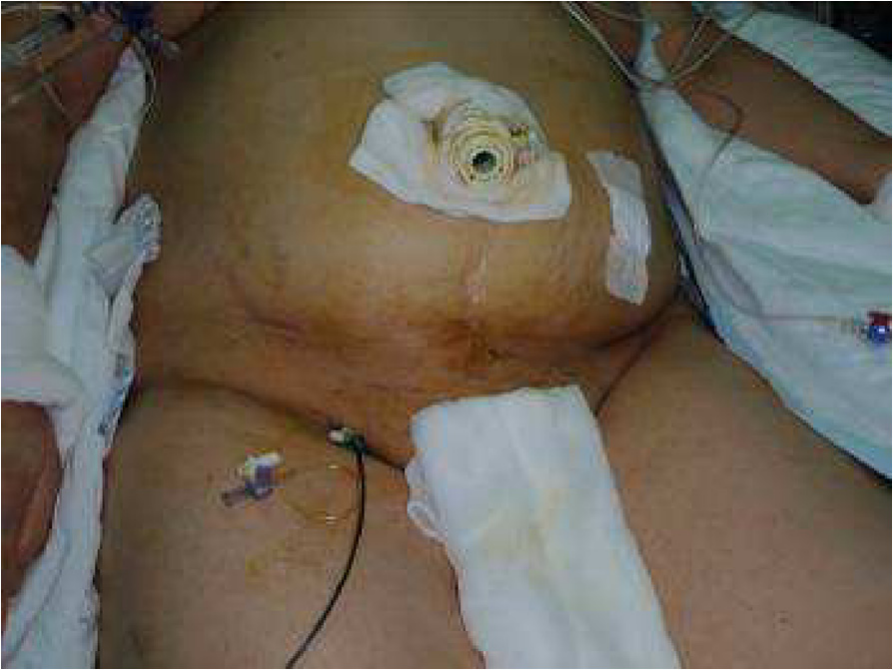
Leaving the laparoscopic port in place after laparoscopic evaluation of the abdomen may enable a quick and easy way of second-look after local thrombolytic therapy.

We have utilized these diagnostic and treatment modalities for AMI in an algorithm that is presented in this paper. The mortality rate in patients without peritoneal signs was 20% (1/5), whilst it was 62.5% (5/8) in patients with peritoneal signs during admission. It is also worth noting that all patients with peritoneal signs presented 24 h after the onset of symptoms. This finding confirms the hypothesis that early diagnosis is extremely important in achieving survival [17,18]. We prefer to use laparoscopy whenever possible. We believe that this may be a good option both in initial and subsequent evaluations. A previously placed laparoscopic port enables a second-look even bedside in the intensive care unit (Figure 4). Second look laparoscopy is one of the mainstays of surgical treatment of AMI for the assessment of intestinal viability, motility, absence of a necrotic segment and to look over anastomosis. Due to the advantages of laparoscopic second look procedure including, shorter operative time and making way to third or even more explorations, we prefer to perform laparoscopic second look. Nevertheless, this algorithm can be used in cases, which have salvageable bowel segments and some time needed for LTT to revascularize the mesenteric circulation.

In conclusion, acute arterial mesenteric ischemia remains one of the most lethal conditions in patients presenting with an acute abdomen. A high index of suspicion is mandatory for diagnosis. CT-angiography combined with early laparoscopic exploration and thrombolytic treatment may have beneficial effects regarding mortality.

## Competing interests

All authours have no conflict of interests.

## Authors’ contributions

FY, OA writting of the manuscript. OA and ISS conception and design of the manuscript, OA and ISS acquisition of data analiying and interpretation of data. ES follow up the patients. AU interventional radiologist speciliast for the radiologicial procuders. HY and MA are the surgens of the cases. MK critical revising and final approval of the manuscript. All authors read and approved the final manuscript.
